# Supplementary material for: Patterns of Intron Gain and Loss in Fungi
Source: PLoS Biol. 2004 Nov 30;2(12):e422. doi: 10.1371/journal.pbio.0020422 (PMC532390; doi:10.1371/journal.pbio.0020422)
Supplement: Table S1 — Also available at http://genes.mit.edu/NielsenEtAl/. (4.3 MB ZIP). [file pbio.0020422.st001.zip › NielsenEtAl/html/390.html]

AN6974.1.NCU06404.1.MG06135.1.FG06209.1


```
 CLUSTAL W (1.82) Multiple Sequence Alignments - Introns Inserted


Sequence 1: NCU06404.1	205 aa
Sequence 2: FG06209.1	203 aa
Sequence 3: MG06135.1	206 aa
Sequence 4: AN6974.1	206 aa
Alignment Length: 208 aa
Number Identitical Residues: 161 aa
Alignment Score (without introns) 6288


MG06135.1 	-MANRNYDVL0IKLLLIGDSGVGKSCCLLRFSEDSFTPSFITTIGIDFKIRTIELDGKRV
NCU06404.1	MSSNRNYDFL0IKLLLIGDSGVGKSCCLLRFSEDSFTPSFITTIGIDFKIRTIELDGKRV
FG06209.1 	MSSNRNYDFL0IKLLLIGDSGVGKSCCLLRFSEDSFTPSFITTIGIDFKIRTIELDGKRV
AN6974.1  	MAGTRNYDFL0IKLLLIGDSGVGKSCCLLRFSEDSFTPSFITTIGIDFKIRTIELDGKRV
          	  ..****.* *************************************************

MG06135.1 	KLQIWDTAGQERFRTITTAYYRGAMGILLVYDVTDERSFNN1IRTWFANVEQHASEGVNK
NCU06404.1	KLQIWDTAGQERFRTITTAYYRGAMGILLVYDVTDERSFNN1IRTWFANVEQHATEGVNK
FG06209.1 	KLQIWDTAGQERFRTITTAYYRGAMGILLVYDVTDERSFNN1IRTWFQNVEQHATEGVNK
AN6974.1  	KLQIWDTAGQERFRTITTAYYRGAMGILLVYDVTDERSFQN1IRTWFSNVEQHASEGVHK
          	***************************************:* ***** ******:***:*

MG06135.1 	ILIGNKCDWEEKRAVSTEQGQALADELGIPFLEVSAKGNINIDKAFYSLASDIKKRTIDT
NCU06404.1	ILIGNKCDWEEKRAVSKEQGQALADELGIPFLEVSAKANINIEEAFFSLANDIKKRIIDT
FG06209.1 	ILIGNKCDWEEKRVVSTEQGQALADELGIPFLEVSAKSNINIDKAFYSLAADIKKRLIDN
AN6974.1  	ILIGNKCDWEEKRAVTTEQGQQLADELGIPFLEVSAKNNINIEKAFYNLASDIKKG-MDS
          	*************.*:.**** *************** ****::**:.** ****  :*.

MG06135.1 	QKDFQNPGSVNVNAGAQGNSAGGM-GGKCC
NCU06404.1	SGKEASGGSSGVNVSD--NSGSGS-GGKCC
FG06209.1 	S-KNDQPAASGVNVND--KSESG--GSKCC
AN6974.1  	S-KPEQTGSQGVSIDNQASGLSGNAGGKCC
          	. .  . .: .*. . .... .* :*.***
```
